# Supplementary material for: DeepSOFA: A Continuous Acuity Score for Critically Ill Patients using Clinically Interpretable Deep Learning
Source: Sci Rep. 2019 Feb 12;9:1879. doi: 10.1038/s41598-019-38491-0 (PMC6372608; doi:10.1038/s41598-019-38491-0)
Supplement: Supplementary file 1 — Supplementary Information [file 41598_2019_38491_MOESM1_ESM.pdf]

DeepSOFA: A Continuous Acuity Score for Critically Ill Patients using Clinically Interpretable Deep Learning

Benjamin Shickel[1], Tyler J. Loftus[2], Lasith Adhikari[3,5], Tezcan Ozrazgat-Baslanti[3,5], Azra Bihorac[3,5,*,+], and Parisa Rashidi[1,4,5,+]

[1]Department of Computer and Information Science and Engineering, University of Florida, Gainesville, FL, 32611, USA.
[2]Department of Surgery, University of Florida, Gainesville, FL, 32611, USA.
[3]Department of Medicine, University of Florida, Gainesville, FL, 32611, USA.
[4]Department of Biomedical Engineering, University of Florida, Gainesville, FL, 32611, USA.
[5]Precision and Intelligent Systems in Medicine (PRISMA[P]), University of Florida, Gainesville, FL, 32611, USA.
*abihorac@ufl.edu
[+]These authors contributed equally to this manuscript.

Model Details

Recurrent neural network (RNN)

For making hourly acuity assessments and mortality predictions, DeepSOFA utilizes a recurrent neural network (RNN), a type of deep learning algorithm that is naturally suited for processing sequential data. The key attribute of an RNN which makes it especially useful for modeling temporal data is its notion of internal memory; as each new time step of a sequence is processed, an RNN updates its hidden state by combining the current step's data with the deep representation of all data it has seen in the past. In its simplest form, the calculation of the RNN internal state is shown in Equation S1, where $U \in \mathbb{R}^{k\,x\,d}$ is the input weight matrix, $V \in \mathbb{R}^{k\,x\,k}$ is the recurrent weight matrix, d is the number of features in the input sequence, k is the tunable dimensionality of the RNN's hidden state, and bias terms are omitted for simplicity.

$$h_t = \sigma(Ux_t + Vh_{t-1}) \qquad \text{(S1)}$$

At each time step t, the corresponding slice of the input sequence $x_t \in \mathbb{R}^d$ is combined with the previous time step's hidden state $h_{t-1} \in \mathbb{R}^k$ via U, V, and a nonlinear activation function σ such as tanh. In practice, h_0 is often initialized to a vector of zeros. Similar to most other current deep learning algorithms, an RNN's weights are trained via backpropagation, a technique for updating a model's internal weights by attributing the final model error to individual parameters based on the flow of gradients from a differentiable loss function.

At any point during processing a sequence, the RNN's current hidden state h_t is the representation of the entire sequence up to the current time. Once the sequence has been fully passed through the RNN, the final hidden state is taken as the deep representation of the entire sequence, which can then be passed to subsequent layers for tasks such as classification. Figure S1 shows two perspectives of the same example recurrent neural network.

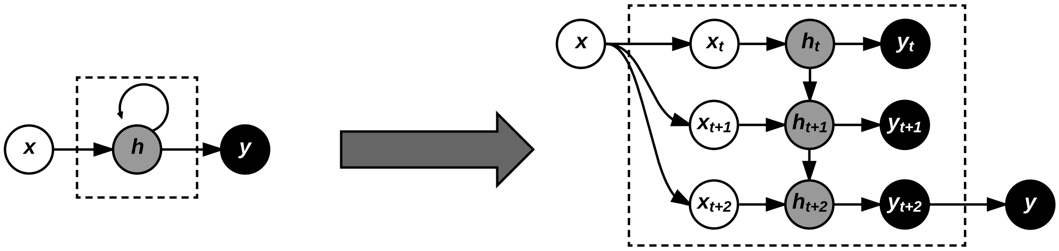

Supplementary Figure S1. Compact (left) and expanded (right) views of a recurrent neural network (RNN). For simplicity, we omit the fully-connected layer(s) typically placed between hidden state h_t and prediction y_t. In this figure, y is taken to be the final prediction of interest for e.g. a traditional sequence classification task, but DeepSOFA uses all time step predictions y_t for real-time acuity assessment and mortality prediction at every hour following ICU admission.

For predicting a classification target (such as DeepSOFA's in-hospital mortality) at a given time t, one would pass the RNN's current hidden state through a final classification layer as in Equation S2, where $W_y \in \mathbb{R}^{Mxk}$ is the output weight matrix, M is the number of available prediction classes (two in DeepSOFA's mortality prediction), k is the tunable dimensionality of the RNN's hidden state, h_t is the RNN's internal hidden state at time t, and the bias term is omitted for simplicity.

$$y_t = W_y h_t \quad \text{(S2)}$$

Gated recurrent units (GRU)

In the previous section, we described the simplest form of a recurrent neural network. In practice, these standard RNNs suffer from what is known as the *exploding gradient problem*, in which repeated multiplications and nonlinear activations involved in updating the hidden state over time result in unstable chain rule-based gradient calculations during the backpropagation process. This issue, combined with the simple form of an RNN's internal memory, often results in non-robust models that are incapable of dealing with longer sequences and at best fail to utilize patterns or dependencies from distant past time steps.

In practice, two popular RNN modifications are typically preferred to the standard RNN, and both attempt to solve the issues above using slightly different techniques. The first RNN variant to be widely adopted is known as *long short-term memory* (LSTM), which augments the traditional RNN with additional weight matrices and gating functions to improve long-range sequential processing. More recently, *gated recurrent units* (GRU) have gained in popularity as a comparable alternative to the LSTM, again involving the introduction of new weights and operations for internally processing a sequence. While research has shown that these two methods result in similar performance for most tasks, DeepSOFA uses GRU networks due to the fewer number of required parameters. Equations S3-S6[1] illustrate the modifications to the traditional RNN from Equation S1, including the introduction of a "reset gate" r_t, an "update gate" z_t, and use of the elementwise multiplication operation $\odot$. Bias terms are omitted for simplicity.

$$r_t = \sigma(W_r x_t + U_r h_{t-1}) \quad \text{(S3)}$$
$$z_t = \sigma(W_z x_t + U_z h_{t-1}) \quad \text{(S4)}$$
$$h'_t = \phi(W_x x_t + r_t \odot (U h_{t-1})) \quad \text{(S5)}$$
$$h_t = (1 - z_t) h_{t-1} + z_t h'_t \quad \text{(S6)}$$

In essence, the introduction of gating mechanisms allows the RNN to become more discretionary in the information it learns and remembers, and the modifications expand the capacity of its internal memory to incorporate important data from potentially long-distant time steps.

Self-attention

[1] https://arxiv.org/pdf/1412.3555.pdf

At each hour during an ICU stay, DeepSOFA makes a mortality probability calculation based on the sequence of EHR measurements available through the current hour. The simplest option for a given hour t would involve passing the GRU's current hidden state h_t through a final output classification layer to produce y_t (Equation S2), the probability of in-hospital mortality given the sequence of measurements encountered thus far. This approach for temporal classification assumes that at a given time t, the entirety of information contained in the input sequence up through time t can be fully represented by the fixed-dimensional vector h_t.

Rather than relying on the most recent hidden state of the GRU for making a prediction, we instead provide a weighted average of all prior hidden states to the final classification layer. The advantages to this approach are twofold. First, the sequence in effect becomes more distilled, by dampening inconsequential time steps and amplifying important ones relative to the final outcome. Second, and most importantly to DeepSOFA, these scalar time step weights can be used for providing clinician insight into the internal reasoning of the GRU's predictions, since larger timestep weights directly influences the averaged hidden state used for prediction and can thus be interpreted as denoting important time steps relative to the outcome of interest.

In the deep learning community, the process of learning scalar time step values to weight a sequence for saliency falls under the umbrella category of an *attention mechanism*, named as such in reference to the act of a model "focusing" on particular pieces of an input rather than its entirety. As is typical, in DeepSOFA we impose the constraint that all attention weights for a given sequence sum to 1 via use of a softmax function over the time dimension, thus encouraging larger weights being placed on the most important time steps of input data. Equations S7 and S8 illustrate a basic attention mechanism involving a global attention vector $W_{att} \in \mathbb{R}^{1xk}$, where relative importance (known in literature as compatibility) of each timestep's hidden state $h_i \in \mathbb{R}^{kx1}$ is calculated via dot product with the global attention vector, and a softmax function over compatibility scores is applied to yield a scalar weight α_i for each timestep $i = 1, 2, \ldots, t$, where the new sequence representation at a given time t is computed as a weighted sum of all preceding hidden states.

$$\alpha_i = \text{softmax}(W_{att} h_i) \qquad \text{(S7)}$$
$$h_t = \sum_{i=0}^{t} \alpha_i h_i \qquad \text{(S8)}$$

Attention mechanisms have garnered increased popularity in recent years, most notably in the field of natural language processing for tasks such as machine translation. In DeepSOFA, we adapt the more recent notion of *self-attention*[2], where rather than learning a global attention vector as in Equation S7, we directly calculate time step compatibilities between hidden time steps themselves. Since DeepSOFA is centered around real-time ICU monitoring, at each hour t during a patient's ICU stay we wish to understand which prior timesteps were most influential in generating the current representation h_t and current prediction y_t. By adopting a variant of self-attention, clinicians are able to understand the interactions between changes in measurements in real-time. Equations S9 and S10 describe DeepSOFA's attention process, where weight matrices $W_Q \in \mathbb{R}^{k\,x\,k}$, $W_K \in \mathbb{R}^{k\,x\,k}$, and $W_V \in \mathbb{R}^{k\,x\,k}$ are learned projections of the same hidden representation $h_i \in \mathbb{R}^{k\,x\,1}$.

$$\alpha_i = \text{softmax}(\,W_Q h_i \,\cdot\, W_K h_i) \qquad \text{(S9)}$$
$$h_t = \sum_{i=0}^{t} \alpha_i W_V h_i \qquad \text{(S10)}$$

At each timestep t during a patient's ICU stay, attention values $\alpha_i \; \forall \; i = 1, .. \, t$ are recalculated to present a current, updated view on the most important time steps influencing mortality prediction. Where traditionally temporal attention values are calculated only after the entire sequence has been seen, to our knowledge DeepSOFA is the first framework to consider real-time self-attention distributions that are updated on-the-fly and only consider currently available EHR information for immediate clinician interpretability.

Model training

The architecture of the DeepSOFA model is relatively straightforward, consisting of (1) an input layer, (2) a single GRU layer, (3) a self-attention layer, (4) a dropout layer, and (5) a final fully-connected output layer for

[2] http://papers.nips.cc/paper/7181-attention-is-all-you-need.pdf

classification. Since DeepSOFA is focused on real-time prediction, we replicate the final mortality target[3] across all input time steps to encourage a correct prediction as early as possible. The model is trained to optimize cross-entropy loss averaged across all hours of an ICU stay (Equation S11), where W_Y is the output weight matrix of the final classification layer, h_t is the attention-weighted sum of available hidden states at each hour $t \; \forall \; t = 1, \ldots T$, and T is the number of hours the patient was in the ICU. Since we apply target replication, y is the same for all time steps t for a given ICU stay.

$$loss = \frac{1}{T} \sum_{t=0}^{T} -[y * \log(W_Y h_t) + (1-y) \log(1 - W_Y h_t)] \qquad \text{(S11)}$$

Model training was terminated when the AUC on the validation set did not increase for 5 epochs. DeepSOFA was specifically trained with a focus on real-time implementation; thus, during training we implemented the dynamic, hourly self-attention updates by copying the input sequence of length T to a $T \; x \; T$ matrix and applying a lower triangular mask for preventing the model from looking ahead to time steps occurring in the future.

Model parameters

In this iteration of our work, we did not perform extensive hyperparameter tuning and simply sought to show that recurrent neural networks, combined with the full continuous scope of a subset of EHR data, outperformed clinical baselines while providing more granular and informative time series patterns to improve ICU bedside monitoring. This initial version of DeepSOFA used a GRU layer with 64 hidden units, 20% dropout, L2 weight regularization of 1e-6, a batch size of 16, and an Adam optimizer.

[3] https://arxiv.org/pdf/1511.03677.pdf

Supplementary tables and figures

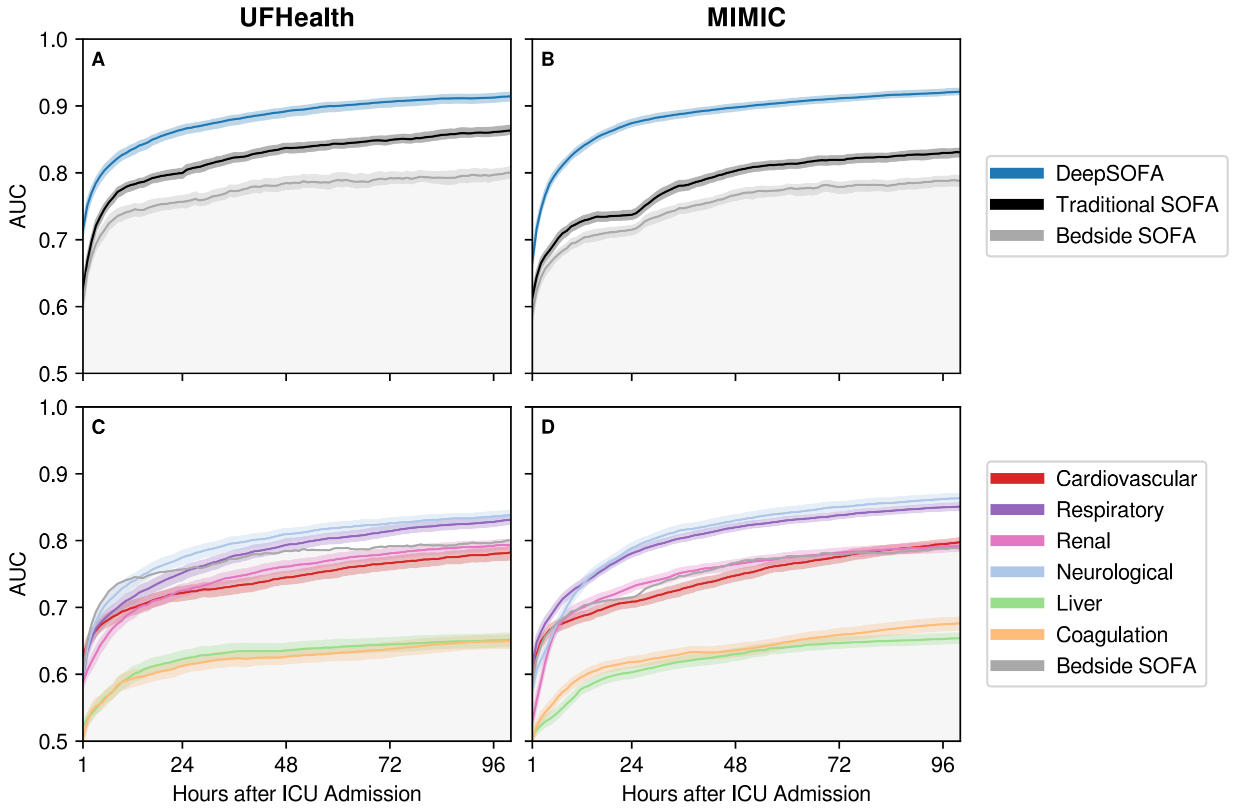

Supplementary Figure S2. (A, B) Internally validated DeepSOFA, Bedside SOFA, and Traditional SOFA score accuracy in predicting in-hospital mortality, expressed as area under the receiver operating characteristic curve (AUC) for the first 100 hours following ICU admission. (C, D) Internally validated DeepSOFA accuracy for individual models corresponding to variable sets derived from SOFA organ system classification for the first 100 hours following ICU admission. Shaded regions represent 95% confidence intervals based on 100 bootstrapped iterations. All internal validation results obtained using 5-fold cross-validation. Columns specify both the development and validation cohort. SOFA: Sequential Organ Failure Assessment.

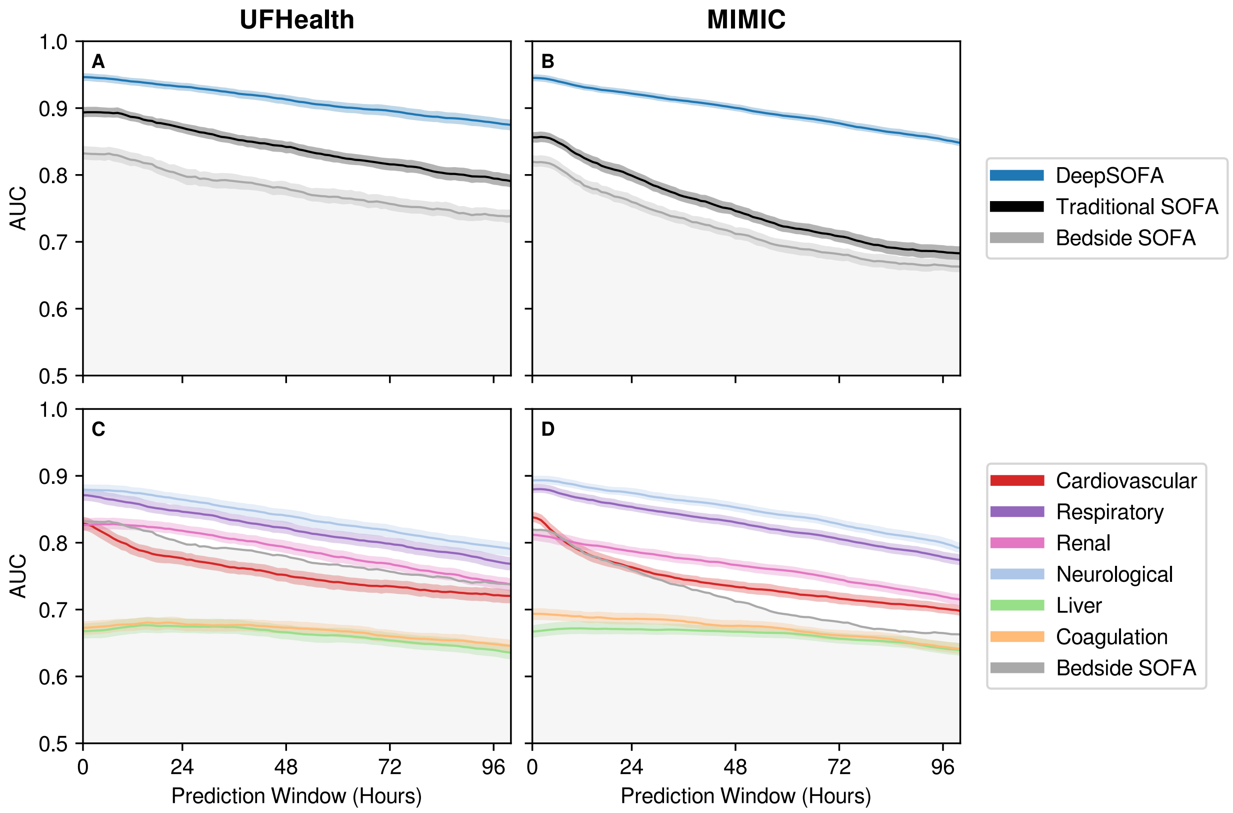

Supplementary Figure S3. (A, B) Internally validated DeepSOFA, Bedside SOFA, and Traditional SOFA score accuracy in predicting in-hospital mortality, expressed as area under the receiver operating characteristic curve (AUC) for 100 hours preceding death or hospital discharge. (C, D) Internally validated DeepSOFA accuracy for individual models corresponding to variable sets derived from SOFA organ system classification for the 100 hours preceding death or hospital discharge. Shaded regions represent 95% confidence intervals based on 100 bootstrapped iterations. All internal validation results obtained using 5-fold cross-validation. Columns specify both the development and validation cohort. SOFA: Sequential Organ Failure Assessment.

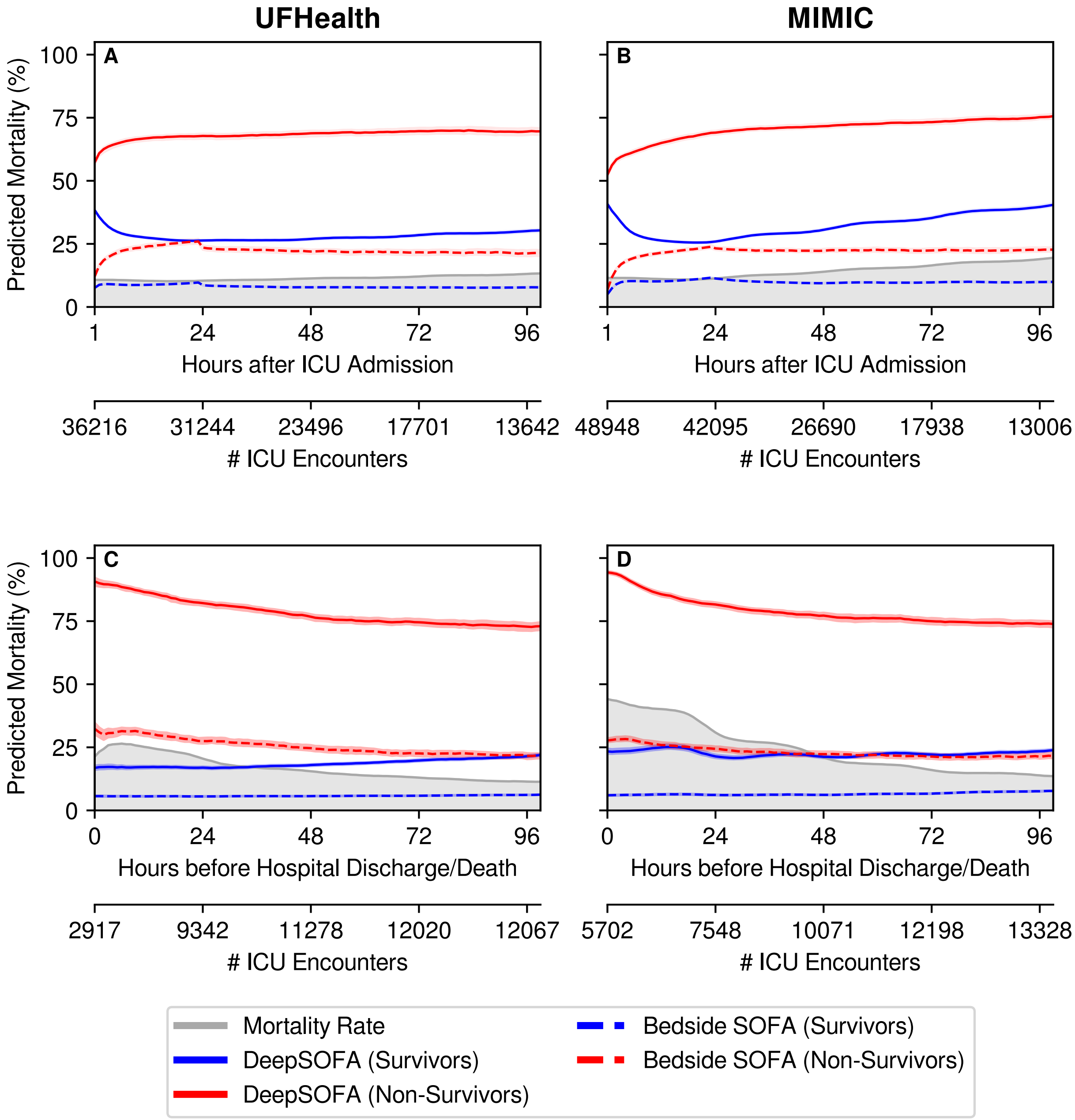

Supplementary Figure S4. Mean predicted mortality probabilities for internally validated DeepSOFA and Bedside SOFA models stratified by outcome. Probabilities shown both for first 100 hours after ICU admission (A, B) and final 100 hours before hospital discharge or death (C, D). Number of ongoing ICU encounters shown below each panel. Shaded regions around each line represent 95% confidence intervals based on 100 bootstrapped iterations. Gray shared area denotes hourly mortality rate for active ICU encounters. All internal validation results obtained using 5-fold cross-validation. Columns specify both the development and validation cohort. SOFA: Sequential Organ Failure Assessment.

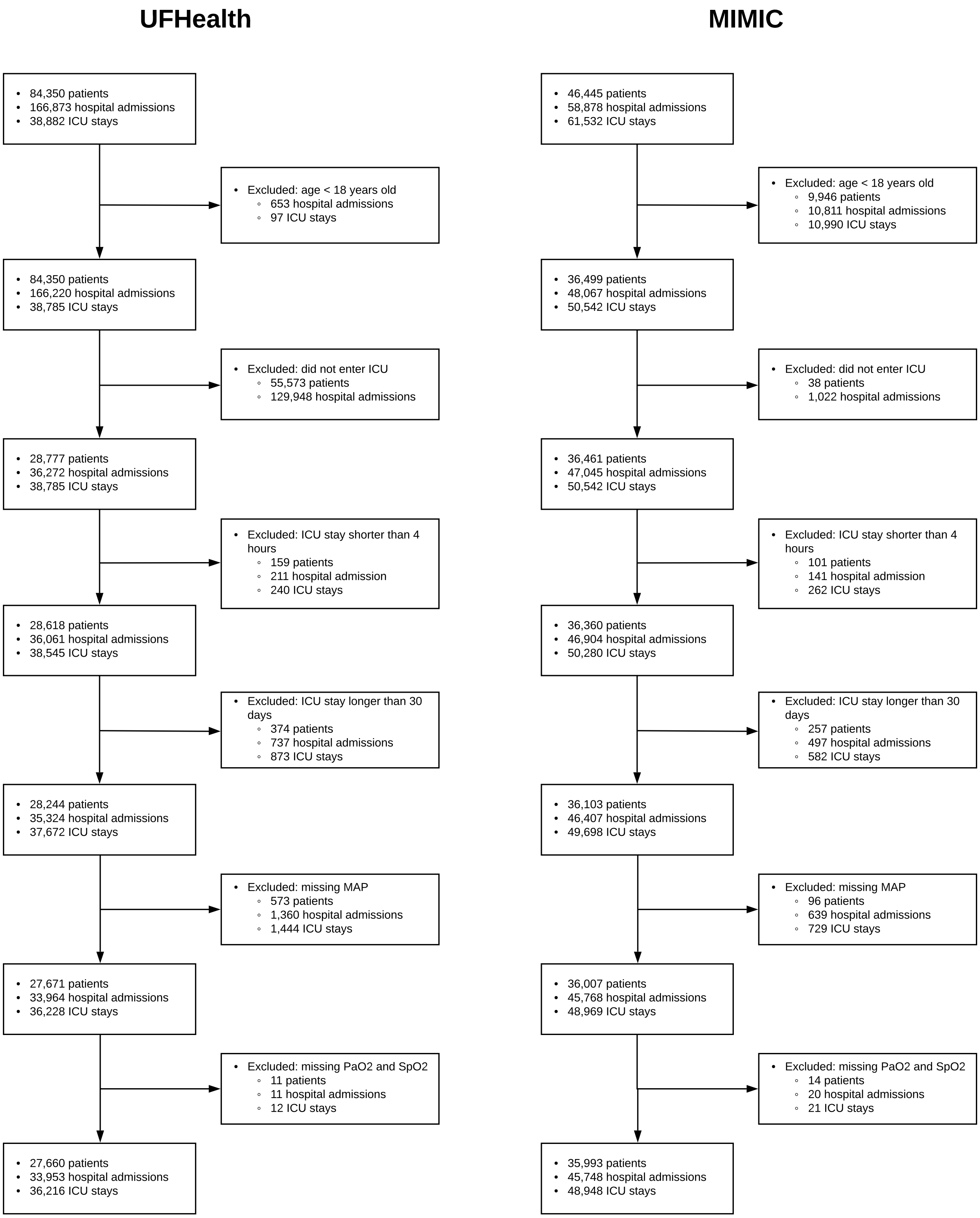

Supplementary Figure S5. Cohort selection and exclusion criteria for both *UFHealth* and *MIMIC* cohorts.

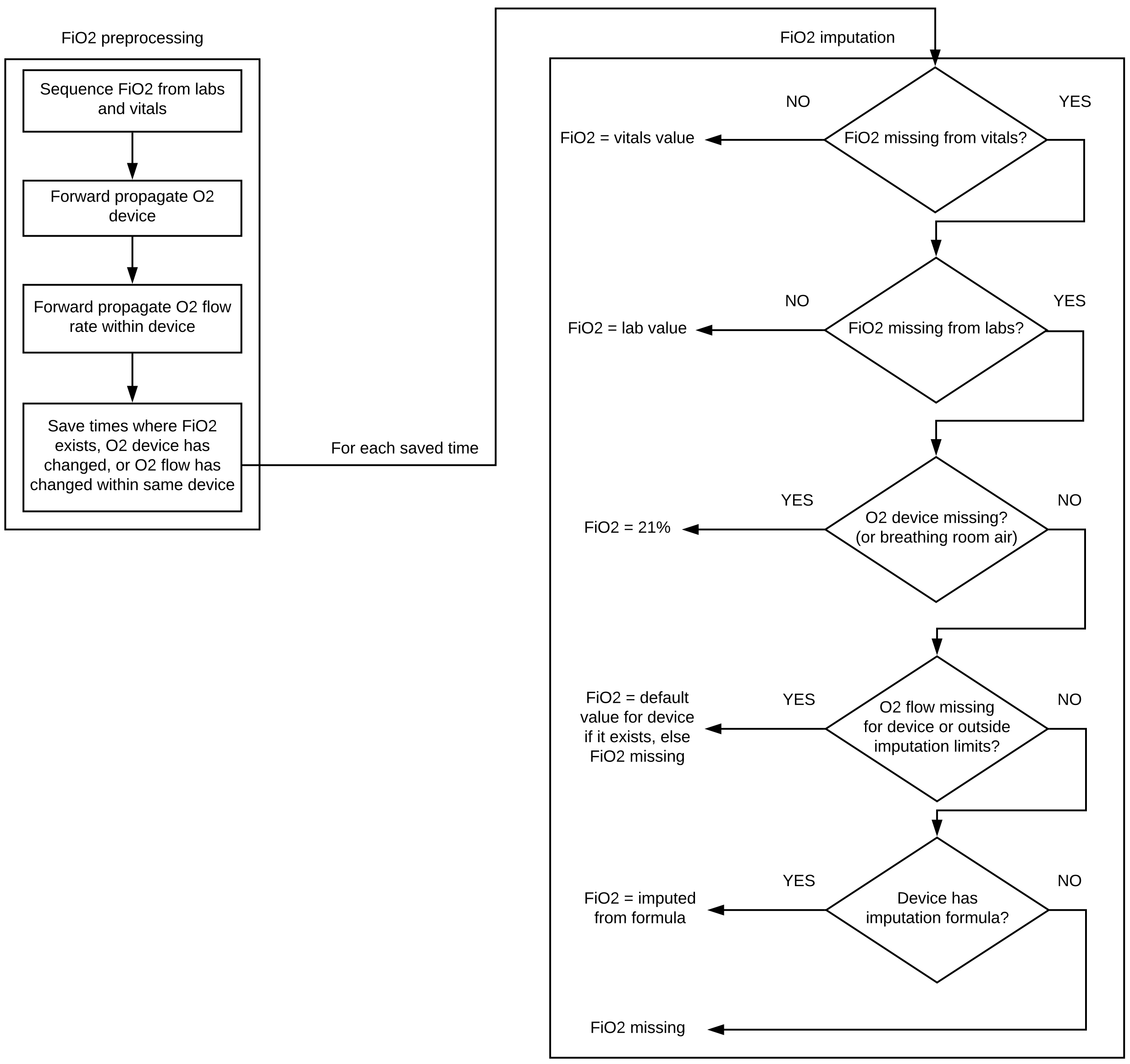

Supplementary Figure S6. Process for FiO2 imputation from existing vitals or laboratory measurements, oxygen delivery device, and corresponding oxygen flow rate.

Device	Default FiO2	O2 limits for imputation [min, max] L/min	FiO2 imputation formula X: O2 flow, L/min	Max imputed FiO2
Aerosol mask	35	[0, N/A]	21 + (X * 4)	60
Nasal cannula	-----	[0, N/A]	21 + (X * 4)	40
High flow nasal cannula	50	[6, 15]	48 + [(X - 6) * 2]	100
Simple mask	-----	[0, 19]	21 + (X * 4)	60
Non-rebreather mask	60	[8, N/A]	80 + [minimum(X - 10, 2) * 10]	100
Venturi mask	35	[4, 8]	26 + [(X - 4) * 2.5]	55
Trach mask	30	-----	-----	-----
CPAP	40	-----	-----	-----
BiPAP	40	-----	-----	-----
Tracheostomy	40	-----	-----	-----
Ventilator	40	-----	-----	-----
Bag valve mask	100	-----	-----	-----
T-piece	40	-----	-----	-----
Transtracheal catheter	40	-----	-----	-----
Blow-by	25	-----	-----	-----
Partial rebreather mask	35	-----	-----	-----
Face tent	25	-----	-----	-----
Oxyimiser	40	-----	-----	-----
Oscillator	80	-----	-----	-----
Oxyhood	35	-----	-----	-----

Supplementary Table S1. Formulas for imputing FiO2 from oxygen delivery device and corresponding oxygen flow rate. If no oxygen flow rate is given, default FiO2 is imputed. If oxygen flow rate is outside specified range, minimum and maximum flow rate is used for imputing FiO2. If formula result is greater than maximum per-device FiO2, the maximum FiO2 is imputed. Abbreviations: CPAP, continuous positive airway pressure; BiPAP, bi-level positive airway pressure.

SOFA component (organ system)	Tradtional SOFA	DeepSOFA	Variables, units of measurement	Non-outlier range [min, max]	Missing values (traditional SOFA)†
**Cardiovascular**	4: Dop > 15 or epi > 0.1 or nor > 0.1<br>3: Dop > 5 or epi <= 0.1<br>2: Dop <= 5 or dob (any dose)<br>1: MAP < 70<br>0: MAP >= 70 and no vasopressors	Uses all available MAP recordings and drug dosing for dop, dob, epi, and nor	Mean arterial blood pressure, mmHg	(0, 300]	SOFA component = 0
			Dopamine, mcg/kg/min	[0, 50]	N/A (normal SOFA calculation)
			Dobutamine, mcg/kg/min	[0, 40]	N/A (normal SOFA calculation)
			Epinephrine, mcg/kg/min	[0, 5]	N/A (normal SOFA calculation)
			Norepinephrine, mcg/kg/min	[0, 15]	N/A (normal SOFA calculation)
**Respiratory**	4: PaO2/FiO2 < 100 w/ MV<br>3: PaO2/FiO2 < 200 w/ MV<br>2: PaO2/FiO2 < 300<br>1: PaO2/FiO2 < 400<br>0: PaO2/FiO2 >= 400	Uses all available PaO2, sPO2, FiO2, and mechanical ventilation status recordings	Fraction of inspired oxygen (FiO2), %	[21, 100]	SOFA component = 0
			Partial pressure of oxygen (PaO2), mmHg	(0, 800]	Use SpO2/FiO2 to PaO2/FiO2 conversion
			Blood oxygen saturation (SpO2), %	(0, 100]	If PaO2 also missing, SOFA component = 0. Otherwise, normal SOFA calculation
			Mechanical ventilation, binary indicator	N/A	N/A (normal SOFA calculation)
**Central nervous system**	4: GCS < 6<br>3: GCS < 10<br>2: GCS < 13<br>1: GCS < 15<br>0: GCS = 15	Uses all available GCS recordings	Glasgow coma scale, score	[3,15]	SOFA component = 0
**Coagulation**	4: Platelets < 20<br>3: Platelets < 50<br>2: Platelets < 100<br>1: Platelets < 150<br>0: Platelets >= 150	Uses all available platelet count recordings	Platelets, count x 10^3/mm^3	(0, 832]*	SOFA component = 0
**Liver**	4: Bilirubin > 12<br>3: Bilirubin >= 6<br>2: Bilirubin >= 2<br>1: Bilirubin >= 1.2<br>0: Bilirubin < 1.2	Uses all available bilirubin recordings	Bilirubin, mg/dL	(0, 50]	SOFA component = 0
**Renal**	4: Creatinine > 5 or urine sum < 200<br>3: Creatinine >= 3.5 or urine sum < 500<br>2: Creatinine <= 2<br>1: Creatinine <= 1.2<br>0: Creatinine < 1.2	Uses all available creatinine and urine recordings	Creatinine, mg/dL	(0, 30]	SOFA component = 0
			Urine, mL	[0, 1095]*	Normal SOFA calculation

Supplementary Table S2. SOFA score, DeepSOFA, variable, and processing definitions. *Threshold values obtained from modified Z-score method (Z > 5)**.** †DeepSOFA model works with all available data, even if some

variables are missing. Abbreviations**:** Dop, dopamine; Dob, dobutamine; Epi, epinephrine; nor, norepinephrine; MAP, mean arterial pressure; MV, mechanical ventilation.

Model	Training Required?	Mortality Probability Prediction at Hour T	AUC Calculation at Hour T
DeepSOFA	Yes	Feed current data sequence from hour 0 to hour T through RNN to get mortality probability prediction.	Get mortality probability predictions at hour T for all ICU encounters in validation cohort. For ICU encounters already completed by hour T, use final mortality prediction. Calculate AUC between mortality probability predictions and true in-hospital mortality labels.
Bedside SOFA	No	Calculate SOFA score for previous 24-hour period (T-24, T]. Consult published mortality rate table relating SOFA score to overall mortality rate. Use mortality rate as patient mortality probability prediction.	
Traditional SOFA		N/A	Calculate SOFA score for previous 24-hour period (T-24, T] for all ICU encounters in validation cohort. For ICU encounters already completed by hour T, use final SOFA score. Calculate AUC between SOFA scores and true in-hospital mortality labels.

Supplementary Table S3. High-level overview of DeepSOFA and SOFA baseline model operation.

		First	24h	48h	72h	96h	Last	Mean
**UFHealth**	DeepSOFA	0.86 (0.86 - 0.87)	0.89 (0.88 - 0.90)	0.91 (0.90 - 0.91)	0.91 (0.91 - 0.92)	0.72 (0.71 - 0.72)	0.95 (0.94 - 0.95)	0.91 (0.90 - 0.91)
	Logistic Regression	0.77 (0.77 - 0.78)	0.80 (0.80 - 0.81)	0.82 (0.81 - 0.83)	0.83 (0.82 - 0.84)	0.61 (0.60 - 0.62)	0.88 (0.87 - 0.89)	0.81 (0.81 - 0.82)
	Random Forest	0.68 (0.67 - 0.69)	0.71 (0.70 - 0.71)	0.72 (0.72 - 0.73)	0.74 (0.73 - 0.75)	0.57 (0.57 - 0.58)	0.81 (0.81 - 0.82)	0.72 (0.71 - 0.73)
**MIMIC**	DeepSOFA	0.87 (0.87 - 0.88)	0.90 (0.89 - 0.90)	0.91 (0.91 - 0.92)	0.92 (0.92 - 0.92)	0.67 (0.66 - 0.67)	0.95 (0.94 - 0.95)	0.91 (0.91 - 0.92)
	Logistic Regression	0.77 (0.76 - 0.77)	0.80 (0.80 - 0.81)	0.82 (0.82 - 0.83)	0.83 (0.83 - 0.84)	0.57 (0.56 - 0.57)	0.87 (0.87 - 0.88)	0.81 (0.80 - 0.81)
	Random Forest	0.67 (0.67 - 0.68)	0.71 (0.70 - 0.71)	0.73 (0.72 - 0.73)	0.74 (0.74 - 0.75)	0.54 (0.54 - 0.55)	0.81 (0.81 - 0.82)	0.72 (0.72 - 0.73)

Supplementary Table S4. Internally validated AUC results for three additional baseline machine learning models compared with DeepSOFA results from main manuscript. Baseline models used 84 aggregate features recalculated at every hour after ICU admission, including the following for each of the 14 SOFA variables: minimum value, maximum value, mean value, standard deviation, first value, and last value. Models trained using aggregate features from entire development ICU stays and evaluated hourly using recalculated features in an expanding window. Results shown for first hour after ICU admission, at time of ICU discharge, mean across all hours, and for 24, 48, 72, and 96 hours after ICU admission. 95% confidence intervals shown in parentheses.

		First	24h	48h	72h	96h	Last	Mean
**UFHealth**	DeepSOFA	0.85 (0.85 - 0.86)	0.89 (0.88 - 0.89)	0.90 (0.89 - 0.91)	0.91 (0.90 - 0.92)	0.70 (0.70 - 0.71)	0.94 (0.94 - 0.95)	0.90 (0.90 - 0.91)
	Logistic Regression	0.76 (0.76 - 0.77)	0.79 (0.79 - 0.80)	0.81 (0.81 - 0.82)	0.83 (0.82 - 0.83)	0.60 (0.59 - 0.60)	0.87 (0.87 - 0.88)	0.80 (0.80 - 0.81)
	Random Forest	0.63 (0.62 - 0.63)	0.65 (0.64 - 0.66)	0.68 (0.67 - 0.68)	0.69 (0.69 - 0.70)	0.53 (0.52 - 0.53)	0.77 (0.77 - 0.78)	0.67 (0.67 - 0.68)
**MIMIC**	DeepSOFA	0.86 (0.85 - 0.86)	0.89 (0.88 - 0.89)	0.90 (0.90 - 0.90)	0.91 (0.91 - 0.91)	0.61 (0.61 - 0.62)	0.94 (0.93 - 0.94)	0.90 (0.90 - 0.90)
	Logistic Regression	0.75 (0.74 - 0.75)	0.79 (0.78 - 0.79)	0.80 (0.80 - 0.81)	0.82 (0.81 - 0.82)	0.58 (0.57 - 0.58)	0.86 (0.85 - 0.86)	0.79 (0.79 - 0.80)
	Random Forest	0.66 (0.66 - 0.67)	0.69 (0.69 - 0.70)	0.72 (0.71 - 0.72)	0.73 (0.72 - 0.74)	0.54 (0.54 - 0.55)	0.81 (0.81 - 0.82)	0.71 (0.70 - 0.72)

Supplementary Table S5. Externally validated AUC results for three additional baseline machine learning models compared with DeepSOFA results from main manuscript. Baseline models used 84 aggregate features recalculated at every hour after ICU admission, including the following for each of the 14 SOFA variables: minimum value, maximum value, mean value, standard deviation, first value, and last value. Models trained using aggregate features from entire development ICU stays and evaluated hourly using recalculated features in an expanding window. Results shown for first hour after ICU admission, at time of ICU discharge, mean across all hours, and for 24, 48, 72, and 96 hours after ICU admission. 95% confidence intervals shown in parentheses.

			First	24h	48h	72h	96h	Last	Mean
UFHealth	DeepSOFA	All	0.86 (0.86 - 0.87)	0.89 (0.88 - 0.90)	0.91 (0.90 - 0.91)	0.91 (0.91 - 0.92)	0.72 (0.71 - 0.72)	0.95 (0.94 - 0.95)	0.91 (0.90 - 0.91)
		First Only	0.87 (0.87 - 0.88)	0.90 (0.89 - 0.91)	0.92 (0.91 - 0.92)	0.92 (0.92 - 0.93)	0.72 (0.71 - 0.73)	0.95 (0.95 - 0.96)	0.91 (0.90 - 0.91)
		Unique Only	0.89 (0.89 - 0.90)	0.92 (0.92 - 0.93)	0.94 (0.93 - 0.94)	0.94 (0.94 - 0.95)	0.73 (0.72 - 0.74)	0.97 (0.97 - 0.97)	0.93 (0.92 - 0.93)
	Traditional SOFA	All	0.80 (0.79 - 0.81)	0.84 (0.83 - 0.84)	0.85 (0.84 - 0.85)	0.86 (0.86 - 0.87)	0.63 (0.62 - 0.63)	0.89 (0.89 - 0.90)	0.85 (0.85 - 0.86)
		First Only	0.81 (0.80 - 0.82)	0.85 (0.84 - 0.86)	0.86 (0.85 - 0.87)	0.87 (0.87 - 0.88)	0.63 (0.62 - 0.64)	0.91 (0.90 - 0.91)	0.85 (0.85 - 0.86)
		Unique Only	0.83 (0.82 - 0.83)	0.87 (0.86 - 0.88)	0.88 (0.88 - 0.89)	0.89 (0.89 - 0.90)	0.63 (0.62 - 0.64)	0.93 (0.92 - 0.93)	0.87 (0.87 - 0.88)
	Bedside SOFA	All	0.76 (0.75 - 0.77)	0.78 (0.78 - 0.79)	0.79 (0.78 - 0.80)	0.80 (0.79 - 0.80)	0.61 (0.60 - 0.62)	0.83 (0.82 - 0.84)	0.79 (0.79 - 0.80)
		First Only	0.77 (0.76 - 0.78)	0.79 (0.79 - 0.80)	0.81 (0.79 - 0.82)	0.81 (0.80 - 0.82)	0.61 (0.60 - 0.62)	0.84 (0.83 - 0.85)	0.80 (0.79 - 0.81)
		Unique Only	0.78 (0.77 - 0.79)	0.81 (0.80 - 0.82)	0.82 (0.82 - 0.83)	0.83 (0.82 - 0.84)	0.61 (0.60 - 0.62)	0.86 (0.85 - 0.87)	0.82 (0.81 - 0.83)
MIMIC	DeepSOFA	All	0.87 (0.87 - 0.88)	0.90 (0.89 - 0.90)	0.91 (0.91 - 0.92)	0.92 (0.92 - 0.92)	0.67 (0.66 - 0.67)	0.95 (0.94 - 0.95)	0.91 (0.91 - 0.92)
		First Only	0.89 (0.88 - 0.89)	0.91 (0.90 - 0.91)	0.92 (0.92 - 0.93)	0.93 (0.93 - 0.94)	0.67 (0.66 - 0.68)	0.96 (0.95 - 0.96)	0.91 (0.91 - 0.92)
		Unique Only	0.91 (0.91 - 0.92)	0.93 (0.93 - 0.94)	0.95 (0.94 - 0.95)	0.96 (0.95 - 0.96)	0.68 (0.67 - 0.69)	0.98 (0.97 - 0.98)	0.94 (0.93 - 0.94)
	Traditional SOFA	All	0.74 (0.73 - 0.74)	0.80 (0.80 - 0.81)	0.82 (0.81 - 0.82)	0.83 (0.82 - 0.84)	0.61 (0.61 - 0.62)	0.86 (0.85 - 0.86)	0.82 (0.81 - 0.82)
		First Only	0.74 (0.73 - 0.74)	0.81 (0.80 - 0.81)	0.83 (0.82 - 0.83)	0.84 (0.83 - 0.84)	0.61 (0.60 - 0.62)	0.86 (0.86 - 0.87)	0.81 (0.80 - 0.82)
		Unique Only	0.76 (0.75 - 0.76)	0.83 (0.83 - 0.84)	0.85 (0.84 - 0.86)	0.86 (0.85 - 0.87)	0.62 (0.61 - 0.63)	0.89 (0.88 - 0.89)	0.83 (0.83 - 0.84)
	Bedside SOFA	All	0.71 (0.71 - 0.72)	0.77 (0.76 - 0.77)	0.78 (0.77 - 0.79)	0.79 (0.78 - 0.79)	0.59 (0.58 - 0.60)	0.82 (0.81 - 0.83)	0.78 (0.77 - 0.79)
		First Only	0.71 (0.70 - 0.72)	0.77 (0.76 - 0.78)	0.79 (0.78 - 0.79)	0.80 (0.79 - 0.80)	0.59 (0.58 - 0.60)	0.83 (0.82 - 0.84)	0.77 (0.77 - 0.78)
		Unique Only	0.73 (0.72 - 0.74)	0.80 (0.79 - 0.80)	0.81 (0.80 - 0.82)	0.82 (0.81 - 0.83)	0.59 (0.58 - 0.60)	0.85 (0.85 - 0.86)	0.80 (0.79 - 0.81)

Supplementary Table S6. Internally validated AUC results for three variants of handling patients with multiple ICU stays. Results shown for first hour after ICU admission, at time of ICU discharge, mean across all hours, and for 24, 48, 72, and 96 hours after ICU admission. “All” denotes using all ICU stays available, and is the setting used in the main manuscript. For patients with multiple ICU stays in their EHR, “first only” denotes removing all but their first ICU stay available, while “unique only” removes these patients altogether, ignoring all of their ICU stays. For each setting, new models were trained and evaluated using the modified datasets. All internal validation results obtained using 5-fold cross-validation. 95% confidence intervals shown in parentheses. Modified ICU stay counts: (1) UFHealth: All = 36,216; First Only = 27,660; Unique Only = 22,229; (2) MIMIC: All = 45,748; First Only = 35,993; Unique Only = 28,445.

			First	24h	48h	72h	96h	Last	Mean
UFHealth	DeepSOFA	All	0.85 (0.85 - 0.86)	0.89 (0.88 - 0.89)	0.90 (0.89 - 0.91)	0.91 (0.90 - 0.92)	0.70 (0.70 - 0.71)	0.94 (0.94 - 0.95)	0.90 (0.90 - 0.91)
		First Only	0.87 (0.86 - 0.87)	0.90 (0.89 - 0.90)	0.91 (0.90 - 0.92)	0.92 (0.91 - 0.92)	0.71 (0.70 - 0.72)	0.95 (0.94 - 0.95)	0.90 (0.90 - 0.91)
		Unique Only	0.89 (0.88 - 0.90)	0.92 (0.91 - 0.92)	0.93 (0.93 - 0.94)	0.94 (0.93 - 0.95)	0.72 (0.71 - 0.73)	0.97 (0.97 - 0.97)	0.92 (0.92 - 0.93)
	Traditional SOFA	All	0.80 (0.79 - 0.81)	0.84 (0.83 - 0.84)	0.85 (0.84 - 0.85)	0.86 (0.86 - 0.87)	0.63 (0.62 - 0.63)	0.89 (0.89 - 0.90)	0.85 (0.85 - 0.86)
		First Only	0.81 (0.80 - 0.82)	0.85 (0.84 - 0.86)	0.86 (0.85 - 0.87)	0.87 (0.87 - 0.88)	0.63 (0.62 - 0.64)	0.91 (0.90 - 0.91)	0.85 (0.85 - 0.86)
		Unique Only	0.83 (0.82 - 0.83)	0.87 (0.86 - 0.88)	0.88 (0.88 - 0.89)	0.89 (0.89 - 0.90)	0.63 (0.62 - 0.64)	0.93 (0.92 - 0.93)	0.87 (0.87 - 0.88)
	Bedside SOFA	All	0.76 (0.75 - 0.77)	0.78 (0.78 - 0.79)	0.79 (0.78 - 0.80)	0.80 (0.79 - 0.80)	0.61 (0.60 - 0.62)	0.83 (0.82 - 0.84)	0.79 (0.79 - 0.80)
		First Only	0.77 (0.76 - 0.78)	0.79 (0.79 - 0.80)	0.81 (0.79 - 0.82)	0.81 (0.80 - 0.82)	0.61 (0.60 - 0.62)	0.84 (0.83 - 0.85)	0.80 (0.79 - 0.81)
		Unique Only	0.78 (0.77 - 0.79)	0.81 (0.80 - 0.82)	0.82 (0.82 - 0.83)	0.83 (0.82 - 0.84)	0.61 (0.60 - 0.62)	0.86 (0.85 - 0.87)	0.82 (0.81 - 0.83)
MIMIC	DeepSOFA	All	0.86 (0.85 - 0.86)	0.89 (0.88 - 0.89)	0.90 (0.90 - 0.90)	0.91 (0.91 - 0.91)	0.61 (0.61 - 0.62)	0.94 (0.93 - 0.94)	0.90 (0.90 - 0.90)
		First Only	0.87 (0.86 - 0.87)	0.90 (0.89 - 0.90)	0.91 (0.91 - 0.92)	0.92 (0.92 - 0.93)	0.61 (0.60 - 0.62)	0.95 (0.94 - 0.95)	0.90 (0.89 - 0.90)
		Unique Only	0.90 (0.89 - 0.90)	0.92 (0.92 - 0.93)	0.94 (0.93 - 0.94)	0.95 (0.94 - 0.95)	0.62 (0.61 - 0.63)	0.97 (0.97 - 0.98)	0.93 (0.92 - 0.93)
	Traditional SOFA	All	0.74 (0.73 - 0.74)	0.80 (0.80 - 0.81)	0.82 (0.81 - 0.82)	0.83 (0.82 - 0.84)	0.61 (0.61 - 0.62)	0.86 (0.85 - 0.86)	0.82 (0.81 - 0.82)
		First Only	0.74 (0.73 - 0.74)	0.81 (0.80 - 0.81)	0.83 (0.82 - 0.83)	0.84 (0.83 - 0.84)	0.61 (0.60 - 0.62)	0.86 (0.86 - 0.87)	0.81 (0.80 - 0.82)
		Unique Only	0.76 (0.75 - 0.76)	0.83 (0.83 - 0.84)	0.85 (0.84 - 0.86)	0.86 (0.85 - 0.87)	0.62 (0.61 - 0.63)	0.89 (0.88 - 0.89)	0.83 (0.83 - 0.84)
	Bedside SOFA	All	0.71 (0.71 - 0.72)	0.77 (0.76 - 0.77)	0.78 (0.77 - 0.79)	0.79 (0.78 - 0.79)	0.59 (0.58 - 0.60)	0.82 (0.81 - 0.83)	0.78 (0.77 - 0.79)
		First Only	0.71 (0.70 - 0.72)	0.77 (0.76 - 0.78)	0.79 (0.78 - 0.79)	0.80 (0.79 - 0.80)	0.59 (0.58 - 0.60)	0.83 (0.82 - 0.84)	0.77 (0.77 - 0.78)
		Unique Only	0.73 (0.72 - 0.74)	0.80 (0.79 - 0.80)	0.81 (0.80 - 0.82)	0.82 (0.81 - 0.83)	0.59 (0.58 - 0.60)	0.85 (0.85 - 0.86)	0.80 (0.79 - 0.81)

Supplementary Table S7. Externally validated AUC results for three variants of handling patients with multiple ICU stays. Results shown for first hour after ICU admission, at time of ICU discharge, mean across all hours, and for 24, 48, 72, and 96 hours after ICU admission. “All” denotes using all ICU stays available, and is the setting used in the main manuscript. For patients with multiple ICU stays in their EHR, “first only” denotes removing all but their first ICU stay available, while “unique only” removes these patients altogether, ignoring all of their ICU stays. For each setting, new models were trained and evaluated using the modified datasets. 95% confidence intervals shown in parentheses. Modified ICU stay counts: (1) UFHealth: All = 36,216; First Only = 27,660; Unique Only = 22,229; (2) MIMIC: All = 45,748; First Only = 35,993; Unique Only = 28,445.
